# Current knowledge, attitude, and patterns of oral contraceptives utilization among women in Jordan

**DOI:** 10.1186/s12905-015-0275-1

**Published:** 2015-12-14

**Authors:** Sanaa K. Bardaweel, Amal A. Akour, Maria-Vanessa Z. Kilani

**Affiliations:** Department of Pharmaceutical Sciences, Faculty of Pharmacy, The University of Jordan, Queen Rania Street, Amman, 11942 Jordan; Department of Biopharmaceutics and Clinical Pharmacy, Faculty of Pharmacy, The University of Jordan, Amman, 11942 Jordan

**Keywords:** Oral contraceptives, Women, Attitude, Knowledge, Behavior

## Abstract

**Background:**

Studies exploring the knowledge, attitude and patterns of OCs use among women in Jordan are lacking. The aim of this study was to assess knowledge, attitude, and patterns of oral contraceptives (OCs) utilization among women in Jordan.

**Methods:**

A face-to-face questionnaire inquiring demographic information and issues related to knowledge and use of OCs was completed by women (*n* = 1571), who have had used OCs at least once in their lifetime. A model was created to assess the effects of knowledge, attitude and previous experience on the patterns of OCs utilization.

**Results:**

Jordanian women exhibited positive attitudes towards OCs efficacy and safety. This positive attitude was approvingly associated with the patterns of use. However, only half of participating women reported that they knew how to use OCs. About 60 % of women received recommendations for OCs use from a physician. Moreover, women’s knowledge about OCs mechanism of action was obtained namely from physician (29.9 %). Side effects were reported in 75.1 % of participating women. Reported side effects were headache (41.2 %), mood swings (35.5 %), irritability (33.5 %) and weight gain (28.7 %). Interestingly, the occurrence of side effects was the main reason for OCs discontinuation.

**Conclusion:**

The study showed that women who have positive attitude toward OCs tend to utilize them more appropriately. However, there is still need for educational programs to enhance knowledge about OCs utilization in Jordan.

**Electronic supplementary material:**

The online version of this article (doi:10.1186/s12905-015-0275-1) contains supplementary material, which is available to authorized users.

## Background

Contraception, the deliberate prevention of pregnancy, can be achieved via several approaches. Some contraceptive methods prevent the release of secondary oocytes and sperms from gonads, others limit sperm access to the mature egg to prevent fertilization, whereas certain contraceptive methods allow fertilization to occur before, ultimately, preventing implantation of an embryo. Except for complete abstinence from sexual intercourse, the incidence of unwanted pregnancies might occur with any of the contraception methods [1–3].

Demographic studies in Jordan indicate that the annual fertility rate is 4.4 per woman [4], with fertility rate being defined as the average number of live births or children per woman [5]. While 1 in 4 women, worldwide, recommence to have a fourth child after three consecutive pregnancies, 50 % of Jordanian women who have had three births would try to get pregnant again within 2 years [6]. The relatively high fertility rate in Jordan with the allied short birth interval between births can be attributed to the lack of knowledge available on possible contraception methods [7, 8]. In addition, several demographic and non-demographic factors have been shown to influence Jordanian women’s beliefs about contraception [6, 9]. Indeed, religion, education, employment and gender of children were among the most prominent factors that influence Jordanian women’s beliefs and behavior towards contraception [10–13].

Oral contraceptives (OCs), are one of the most prevalent forms of reversible contraceptive methods used among women of reproductive age worldwide [14, 15]. In Jordan, OCs are the second most popular method of contraception preceded by the intrauterine devices [9]. Unfortunately, Jordan Pharmacy law has no regulations to restrict prescription medications use, hence, OCs can be obtained from community pharmacies without prescription. In spite of their widespread utilization in Jordan, several misconceptions dominate common knowledge of Jordanian women regarding OCs. In 1996, Farsoun et al. identified decisive hurdles to the use of modern contraceptive methods in Jordan. Serious side effects due to OCs, such as cancer, back pain, headaches, dizziness, hair loss, weight gain, and infertility, were all linked to Jordanian women’s beliefs about OCs.

During the last decade, augmented emphasis on the use of OCs to prevent pregnancy has been practiced by media to promote their utilization among Jordanian women. The later also comes along with education to enhance their awareness of safe and effective use of OCs. In spite of that, no studies have taken place since the last two decades to address this issue. Studies exploring the knowledge, attitude and patterns of OCs use among women in Jordan are, therefore, needed. In fact, Jordanian women could potentially benefit from increased information and advice on OCs to ensure better and regulated use that is compatible with their therapeutic purpose. Hence, the aim of this study was to describe four different aspects of OCs utilization among women in Jordan; patterns of use, attitude toward use, knowledge and previous experience. The study also aimed to determine where women acquire advice and whom they consult regarding their OCs utilization.

## Methods

Ethical approval to perform the study was obtained from the Scientific Committee at the Deanship of Scientific Research at The University of Jordan. Participants’ information remained confidential and within the institution. Verbal informed consent to participate in the study was obtained based on a standard written statement.

Jordanian women who, were married, able to give informed consent, and used OCs at least once in their lifetime, were asked to participate in the study. Many misconceptions, like fear of side effects, especially among unmarried females are strongly dominant among Jordanian women towards the use of contraceptives. Indeed, very small percentages of OCs users are unmarried females and thus this sample criterion was designed to suit the sociocultural characteristics of the Jordanian community.

Data were collected from women living in different districts of Jordan. Selection was performed via a convenient non-random sampling technique from women who visit community pharmacies, fertility, obstetrics and gynecology outpatient clinics in the capital city of Jordan, Amman. The study took place between March 2013 and December 2013.

The study questionnaire was structured based on initial discussions with women, using OCs, and health professionals (Additional file 1). Furthermore, the questionnaire was validated by a committee whose members were health professionals, consisting of three family medicine physicians, a pharmacist, and a nurse. The questionnaire was written in English and then translated into Arabic. Both versions of the questionnaire were checked by three members of the public with no medical background.

The questionnaire was administered by well-trained administrators on each site. The principle investigator coordinated the logistic aspects of questionnaire distribution, data collection, and following responses. The questionnaire gathered data about four aspects of OCs use; patterns of use, attitude toward use, knowledge and previous experience. This was based on the hypothesis that three predictor variables, knowledge, attitude and previous experience, can influence the patterns of use of OCs. Knowledge was measured through direct questions inquiring whether participants know how OCs manifest their action in the body, how to self-administer OCs, how to maintain the efficacy of OCs, and if OCs have potential drug-drug interactions. Secondly, attitude was assessed by asking the participants if they prefer to use OCs, if they think OCs are effective, or safe, or if they fear side effects. Previous experience was measured via asking whether OCs were successful in preventing pregnancy, or if side effects ensue that women had to stop their pill use (refer to Table 2 for detailed description of items). Patterns of use, which is the outcome variable, was evaluated by examining the purpose of pill use, and whether or not women had received doctor’s consultation or prescription before using the pill.

### Data analysis

Data were coded, entered and analyzed using Statistical Package for Social Sciences program (SPSS) database for Windows, version 17 (SAS Institute, Cary, NC). The analysis of answers involved descriptive quantitative statistics e.g. frequency and percentage. Chi-square and Fisher exact tests were used to test for significant association between groups.

A pattern of use, i.e. the outcome variable, was converted into a categorical variable: the sum of three variables was obtained (pill used for birth control purposes, doctor consultation obtained, and pill was prescribed). Those who scored < two were defined as inappropriate users, while those who scored > two were considered as appropriate users. In the same way, the predictors were decoded into categorical variables. Attitudes were considered positive if a score of more than 3 out of 5 was achieved, good knowledge and experience is defined as a score more than 3 out of 5 and 4 out of 6; respectively. To assess the relationship between knowledge, attitude, previous experience and the patterns of use, logistic regression was utilized. All hypothesis testing was two-sided, with a probability value of 0.05 deemed as significant.

## Results

### Study demographics

In this study, 2000 questionnaires were distributed among Jordanian women who use oral contraceptives, and thus were included in the study, and data were collected from 1571 women (response rate 78.5 %). Table 1 describes the demographic characteristics of the study sample. Participants’ ages ranged between 18 and 50 years old. The majority of women has been married for more than 5 years (≈80 %), and about 60 % of the participating women has three or more children. More than half of the study sample included housewives (55.7 %) living in the capital of Jordan “Amman” (68.2 %). One third of the sample has high school education, and about the same percentage has an undergraduate education. Two thirds of the sample was of low to intermediate (up to 1000 JD) monthly income.

**Table 1:** Demographic characteristics of women using oral contraceptives in Jordan, 2013

DESCRIPTIVE PARAMETER	N (VALID PERCENT %)
Age in years (*n* = 1564)	
18–25	213 (13.6)
26–30	294 (18.8)
31–35	250 (16.0)
> 35	807 (51.6)
Education (*n* = 1543)	
Primary school	115 (7.50)
High school	501 (32.5)
Community college	288 (18.7)
Undergraduate	542 (35.1)
Graduate	97 (6.30)
Occupation (*n* = 1530)	
Student	71 (4.6)
Employed	532 (34.8)
Housewife	875 (55.7)
Retired	52 (3.3)
Residence city (*n* = 1538)	
Amman	1071 (68.2)
Irbid	69 (4.4)
Al-Zarqa’	271 (17.3)
Others	127 (13.2)
Monthly income of the family (*n* = 1542)	
< 1000 JOD	1055 (68.4)
> 1000 JOD	487 (31.6)
Marriage duration (*n* = 1462)	
< 1 year	26 (1.8)
1–2 years	73 (5.0)
2–5 years	212 (14.5)
More than 5 years	1151 (78.8)
Number of previous pregnancies (*n* = 1376)	
≤ 2	305 (22.2)
3–4	461 (33.5)
> 4	545 (39.6)
Nulliparous	65 (4.7)
Number of children (*n* = 1428)	
≤ 2	505 (35.4)
3–4	544 (38.1)
More than four	379 (26.5)
Number of abortions (*n* = 1448)	
≤ 2	1317 (91.1)
3–4	97 (6.7)
> 4	334 (2.3)

### Patterns and attitudes

The pill utilization pattern, attitude towards pills use and knowledge were described in Table 2. The pill utilization pattern showed a positive trend. Indeed, more than half of women used pills for birth control purposes; i.e. contraception. In addition, more than 75 % of women received doctor consultation and prescription before usage. Although OCs utilization pattern was considered appropriate among participating women, the attitude toward using OCs was neutral. Nearly about 60 % of women prefer using OCs as a method for contraception. In spite of neutral attitude toward safety, participants had a positive attitude toward efficacy. In fact, more than 80 % of the study population believes that pills are effective. Nonetheless, 74.7 % were concerned about side effects, namely, hormonal disturbances (Table 2).

**Table 2:** Oral contraceptives utilization pattern, attitude and knowledge

PARAMETER	N (VALID PERCENT %)
PILL UTILIZATION PATTERN	
Purpose of Use (*n* = 1571)^a^	
Birth control	1252 (79.7)
Stop menstruation	232 (14.8)
Others	160 (10.2)
Doctor consultation before use (*n* = 1553)	
Yes	1282 (82.5)
No	271 (17.5)
Pills were prescribed (*n* = 1538)	
Yes	1198 (77.9)
No	340 (22.1)
ATTITUDE TOWARD PILL USE	
Prefer use of pills as method of contraception (*n* = 1519)	
Yes	901 (59.3)
No	618 (40.7)
Reasons for preferring pills (*n* = 1571)	
Ease of use	410 (26.1)
Availability	192 (12.2)
Effectiveness	252 (16.0)
Suitability for body	196 (12.5)
Safety	147 (9.4)
Others	374 (23.8)
Think pills are safe (*n* = 1519)	
Yes	899 (59.2)
No	620 (40.8)
Think pills are effective (*n* = 1506)	
Yes	1250 (83.0)
No	256 (17.0)
Fear of pills’ side effects	
Yes	1148 (74.7)
No	388 (25.3)
Believe pills can cause^a^	
Breast Cancer	197 (12.5)
Uterine Cancer	172 (10.9)
Infertility	201 (12.8)
Hormonal Disturbances	738 (47.0)
None	518 (33.0)
PREVIOUS EXPERIENCE WITH PILLS	
Pills were effective (*n* = 1296)	
Yes	1078 (83.2)
No	218 (16.8)
Ever had side effects (*n* = 1561)	
Yes	1172 (75.1)
No	389 (24.9)
Ever stopped pills (use alternative method) (*n* = 1406)	
Yes	903 (64.2)
No	503 (35.8)
Reason for stopping pills^a^	
Side effects	546 (34.8)
Ineffectiveness	66 (4.2)
Difficulty of use	241 (15.3)
Others	117 (5.62)
Pills recommended by^a^	
Husband	154 (9.8)
Family	190 (12.2)
Neighbors	91 (5.8)
Physician	954 (60.7)
Pharmacist	83 (5.3)
Media	96 (6.1)
Others	101 (6.5)
KNOWLEDGE	
How to use pill (*n* = 1497)	
Yes	810 (54.1)
No	687 (43.7)
Source of knowledge about mechanism of pills’ action	
Physician	470 (29.9)
Pharmacist	93 (5.9)
Media	111 (7.1)
Others	258 (16.4)
If medications can counteract the pill efficacy (*n* = 1494)	
Yes	224 (15.0)
No	1270 (85.0)
Antibiotics can counteract the pill (*n* = 1492)	
Yes	265 (17.8)
No	1227 (82.2)
Received instructions how to use the pill (*n* = 1509)	
Yes	1122 (74.4)
No	387 (25.6)
Received instructions to maintain pill efficacy (*n* = 1490)	
Yes	680 (45.6)
No	810 (54.4)

^a^This question could have had more than one of the answers; hence, the cumulative frequency is more than 100 %

### Previous experience

**Fig. 1:**
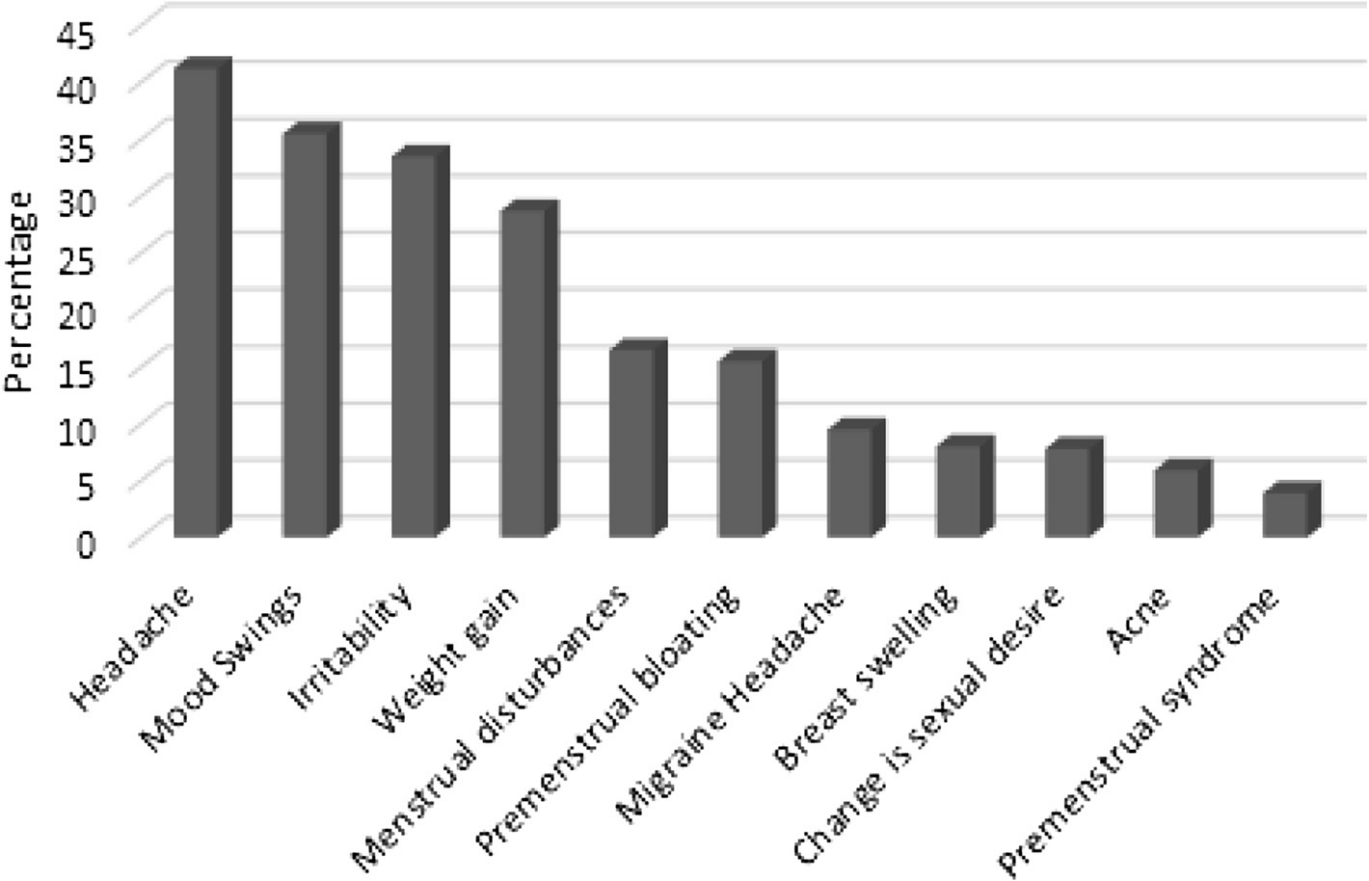
Side effects encountered by women using oral contraceptives

**Fig. 2:**
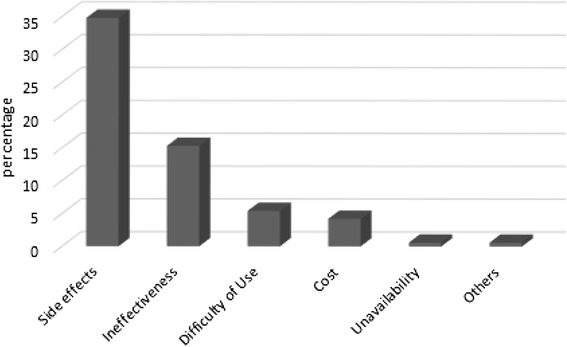
Reasons for oral contraceptives cessation for women in Jordan

About 83 % of participating women stated that OCs were effective; however, side effects were also reported in 75.1 % of the study population. Reported side effects were headache (41.2 %), mood swings (35.5 %), irritability (33.5 %) and weight gain (28.7 %) (Fig. 1). As expected, the incidence of side effects was the main reason for OCs cessation and the utilization of alternative contraception methods (Fig. 2). Cost and unavailability of OCs only had minor effect (0.12 %) the decision to stop pills, which is considered as a good indicator of the health care system in Jordan.

### Knowledge

Although (82.5 %) of the participating women received consultation from the physician, only half of women knew how to use OCs (Table 2). Surprisingly, very little consultation was obtained from pharmacists (Table 2). Only 15 % of the participating women knew that certain medications could decrease OCs efficacy, and consequently, about the same percentage reported the knowledge that antibiotics may deactivate OCs. Nevertheless, 74.4 % of women got instructions for OCs use from physicians.

As stated in the “methods” section, a model was created to study the effect of knowledge, attitude, and previous experience on patterns of OCs utilization (Table 3). Binary logistic regression revealed that the model has 84.8 % predictive power and it was significant (*p*-value =0.011). R^2^ was 0.017, which means that the independent variables can explain 17 % of variance in the dependent variable. Only attitude was significantly associated with appropriate utilization (OR = 1.75, *p* < 0.002). Women with positive attitude are 75 % more likely to use OCs appropriately. Unfortunately, knowledge and previous experience did not have a significant effect on use patterns. Therefore, the effect of each individual item on the outcome variable was evaluated separately.

**Table 3:** Binary logistic regression of knowledge, attitude and previous experience with utilization of oral contraceptives

Covariate	% positive	*p*-value	OR	95 % CI of OR
Knowledge	90.8	0.237	0.675	0.352–1.294
Attitude	40.3	0.002	1.751	1.227–2.500
Previous Experience	43.1	0.244	0.809	0.566–1.156

As previously mentioned, patterns of use, the outcome variable, was generated by the sum of three items (pill use for contraception, physician consultation and pill prescription), with those scoring more than 2 considered as appropriate users, i.e., showing positive utilization patterns. Chi-square test, to study the correlation between individual items of the questionnaire and patterns of use, was performed (Table 4). Interestingly, 82.1 % of women were considered as “appropriate users”. No significant differences in OCs appropriate utilization were found according to demographic variables, i.e. age, education, outcome, etc. The percentage of appropriate pill users was significantly higher in women who prefer using the pill as contraception method compared to those who do not (84.9 % vs. 79.3 %; respectively; *p* = 0.005). In addition, when women prefer OCs because they think they are effective or suitable for their body, they tend to use the pill more appropriately (87.2 % vs. 81.5 % {*p* = 0.000} and 94.8 vs. 80.6 {*p* = 0.029}); respectively. Those who had good attitude about OCs efficacy and safety tend to be appropriate users; among those who think OCs are effective, 84.0 % were appropriate users compared to those who did not (75.7 %); *p* = 0.002. Likewise, women who think OCs are safe, are more likely to be appropriate users (85.6 % vs. 78.6 %). Moreover, if OCs were indeed effective, appropriate use was more likely (86.0 vs. 77.5 %; *p* = 0.002). Furthermore, there was a correlation between the source of recommendation of pill use and the patterns of use. If a physician recommends the pill, the percentage of appropriate users was higher (97.6 % vs. 58.3 %). If the pill was recommended by other sources, however, the trend is reversed. When OCs are recommended by family, neighbors, pharmacists or media, the proportion of appropriate users will be less (52.7 % vs. 86.4 %, 42.2 % vs. 84.9 %, 58.3 % vs. 97.6 % and 61.5 % vs. 83.7 %); respectively.

**Table 4:** Pearson correlation of individual characteristics with oral contraceptives utilization

Covariate	Chi-square	df	*p*-value
Prefer using pills as a method of contraception	7.944	1	0.005
Reason for preference is suitability for body	23.504	1	0.000
Reason for preference is effectiveness of pill	4.757	1	0.029
Thinks pills are effective	10.060	1	0.002
Thinks pills are safe	12.033	1	0.001
Thinks pills cause infertility	4.444	1	0.035
Pills were effective	9.775	1	0.002
Knowledge how pill works	43.110	1	0.000
Source of knowledge about mechanism of pills’ action is physician	92.967	1	0.000
Source of knowledge about maintenance of pill efficacy is physician/pharmacist	62.821	1	0.000
Source of knowledge about use of pill is physician/pharmacist	115.408	1	0.000
Pill was recommended by physician	386.358	1	0.000
Pill was recommended by pharmacist	64.435	1	0.000
Pill was recommended by family	127.275	1	0.000
Pill was recommended my neighbors	106.715	1	0.000
Pill was recommended by media	29.384	1	0.000
Ever suffered from side effects	6.747	1	0.009
Side effects as reason for stopping the pill	5.856	1	0.016
Ever suffered from headache	8.141	1	0.004
Ever suffered from breast swelling	8.090	1	0.004

Interestingly, even if women suffered from side effects, the tendency of appropriate use increases (83.9 vs 78.0 %), specifically if the side effect experienced was headache (85.7 % vs 80.1 %) or breast swelling (91.6 % vs 91.8 %). However, the percentage is less if women believe that pills could cause infertility (77 % vs. 83.2 %). This result implicate that the aforementioned side effects would be experienced despite the appropriate use of OCs.

When women are more knowledgeable about the mechanism OCs action, they will have a higher probability of appropriate use (88.5 % vs. 75.4 %); especially if the physician was the source of knowledge (96.7 % vs. 76.2 %) and if the physician/pharmacist informed them how to maintain pill efficacy (91.1 % vs. 75.3 %) and how to use the pill (88.8 % vs. 64.4 %).

## Discussion

This is the first large cross-sectional study to evaluate oral contraceptives (OCs) utilization patterns among Jordanian females of a wide age range (18–50 years). This study used rigorous comprehensive and face-to-face interview. Furthermore, the later was filled in a reliable, valid questionnaire. In addition, this study was designed to unravel the attitude of Jordanian female population specifically towards OCs and, therefore, promote education programs that could preferentially enhance OCs utilization in Jordan.

In this study, Jordanian women showed a good pattern of OCs utilization; the majority of them were using OCs for birth control purposes, and they received prescriptions and consultations from physicians. Interestingly, no significant differences in OCs utilization pattern were found according to demographic variables, i.e. age, education, outcome, etc. In addition, Jordanian women have positive attitudes regarding OCs efficacy and safety. However, only half of women self- reported that they knew how to use OCs, and only few of them are actually aware of medications that can decrease the pill efficacy. Most women received recommendations for OCs use from a physician. Moreover, the physician was the main source of knowledge for OCs. Unfortunately, the contribution of other healthcare professionals, such as pharmacists and nurses, was minor. All items of knowledge were positively correlated with the use patterns.

Surprisingly, women who suffered from side effects were more likely to be appropriate users. This might be attributed to the fact that the side effects, mostly experienced by these women, were minor in nature, ranging between headache, mood swings and irritability, and consequently women were least concerned about them. On the other side, suffering from side effects was the main reason women to stop OCs and switch to an alternative contraception method. Collectively, the positive attitude toward use was highly correlated with the use patterns.

Historically, there was a negative attitude toward the use of OCs in middle-eastern societies, and Jordan was not an exception [8, 11, 12, 16, 17]. Many misconceptions, like fear of side effects and underestimating efficacy, prevented women from OCs use [8]. In 2004, Kirdli et al.[18] showed that positive attitude and beliefs were among the factors that contributed to Jordanian women’s intention to use OCs, which complies with the results from our study. Since then, many educational programs were implemented to enhance women’s attitudes and knowledge on the rationale use of OCs. Consequently, we expect a leap with regard to these aspects within the last 10 years.

In addition, individual [19], sociocultural [20], institutional and political [21] factors that are substantial in women’s preferences related to OCs use have been investigated in different countries. Among the factors that have specifically captured the attention of healthcare researchers is the attitude of women towards OCs [22, 23]. Unfortunately, most studies were designed to investigate attitudes toward contraceptives in general [24]. Limited number of studies has been designed to study attitudes toward specific contraceptive methods such as OCs. This study was designed to unravel the attitude of Jordanian female population towards OCs and, therefore, promote education programs that could preferentially enhance OCs utilization in Jordan. In addition, no studies have ever described the knowledge of Jordanian population about rationale pill use, and patterns of utilization. The aim of this study was, therefore, to describe four different aspects of pill utilization including patterns of use, attitude toward use, knowledge and previous experience.

Our findings imply that more education programs about OCs use are still warranted. Although more than half of participating women self-reported that they knew how to use OCs, few women could identify medications that interact with OCs, or interfere with their efficacy. Less than 50 % received instructions on how to maintain the efficacy of OCs to prevent pregnancies. For most cases, physicians were the main source of knowledge. Hence, there is a need to increase the contribution of other healthcare professionals such as nurses and pharmacists into the education process, especially about the rationale use of OCs and how to maintain their efficacy. Furthermore, it is essential to enhance the role of other accessible sources of knowledge such as media, workshops, focus groups and internet, in the improvement of women awareness toward OCs use as a critical part of the family planning continuum.

One of the limitations of this study, which is inherent to cross-sectional studies, that it can only assess association between variables at one point of time. In addition, a convenient non-random sampling technique was adopted which, at least in part, explains that most of women in the study resides in Amman, the capital of Jordan. It will be therefore hard to generalize our findings to residents in other cities of Jordan.

## Conclusions

In conclusion, this study revealed an improvement of utilization patterns and attitudes towards OCs among Jordanian women over the last 10 years. However, there is still a gap in the knowledge about how to use OCs and how to maintain their efficacy. The study showed that women who has positive attitude toward OCs use tend to utilize them more appropriately. Educational programs provided via various healthcare professionals and other sources can enhance women’s knowledge about the rationale OCs utilization, and thus maximize beneficial effects and reduce side effects. The later will increase the tendency of having positive experience and thus positive attitudes toward OCs usage. It would be crucial for future researchers to investigate attitudes and knowledge of OCs use in women who have never used OCs and compare it to those who have had used them, as well as compare it toward that of using alternative contraceptive methods, such as intrauterine devices, and transdermal batches. Moreover, studying the effect of various educational programs on the appropriate utilization of OCs is warranted.

## Additional file

Additional file 1:
**The usage of contraceptive pills in birth control by a sample of Jordanian women.** (PDF 195 kb)
