# Supplementary material for: Current knowledge, attitude, and patterns of oral contraceptives utilization among women in Jordan
Source: BMC Womens Health. 2015 Dec 14;15:117. doi: 10.1186/s12905-015-0275-1 (PMC4677435; doi:10.1186/s12905-015-0275-1)
Supplement: Additional file 1: — The usage of contraceptive pills in birth control by a sample of Jordanian women. (PDF 195 kb) [file 12905_2015_275_MOESM1_ESM.pdf]

<u>The usage of contraceptive pills in birth control by a sample of Jordanian women (August 2013)</u>

1. Age □ 18-25 □ 26-30 □ 31-35 □ 36-40 □ 41-45 □ more than 46

2. Education

□ Primary □ Secondary □ Community college □ Undergraduate □ Postgraduate

3. Occupation □ Student □ Employee at public sector □ Employee at private public

□ Business owner □ Housewife □ Retired

4. Residence □ Amman □ Irbed □ Zarqaa □ Salt □ Ma'an □ Madaba □ Tafileh □ Mafraq

□ Aqaba □ Karak □ Jarash □ Other cities in Jordan

5. Nationality □ Jordanian □ Not Jordanian

6. Monthly income of the family □ Less than 1000 JD □ More than 1000 JD

7. Number of members who benefit from the monthly income

□ 1-2 □ 3-5 □ 6-10 □ More than 10

8. Marriage period □ Less than 1 year □ 1-2 years □ 2-5 years □ More than 5 years

9. Any previous pregnancies? □ Yes □ No

10. If the answer to the previous answer was yes, how many? □ 1 □ 2 □ 3 □ 4 □ More than 4

11. Do you have children? □ Yes □ No

12. If the answer to the previous answer was yes, how many? □ 1 □ 2 □ 3 □ 4 □ More than 4

13. Has there been any abortions? □ Yes □ No

14. If the answer to the previous answer was yes, how many? □ 1 □ 2 □ 3 □ 4 □ More than 4

15. Have you ever used contraceptive pills? □ Yes □ No

16. If the answer to the previous answer was yes, for what purpose did you use them?

□ Birth control □ To stop menstrual cycle □ Other

17. Have you consulted a doctor before using the pills? □ Yes □ No

18. Were pills dispensed with prescription? □ Yes □ No

19. Do you prefer using contraceptive pills on other contraceptive methods? □ Yes □ No

20. If the answer to the previous answer was yes, what is the reason?

□ Easiness of use □ Availability □ Effectiveness □ Suits body type □ Safe

21. Do you think that contraceptive pills are effective? □ Yes □ No

22. Do you think that contraceptive pills are safe? □ Yes □ No

23. Are you afraid of the side effects of the pills? □ Yes □ No

24. Do you think that contraceptive pills are related to any of the following diseases?

□ Breast cancer □ Uterine cancer □ Infertility □ Hormonal turbulence □ None

25. Did you suffer any side effects from the pills? □ Yes □ No

26. If the answer to the previous answer was yes, what side effects did you manifest?

□ Weight gain □ Mood swings □ Headache □ Migraine □ PMS □Premenstrual bloating

□ Irritability □ Breast swelling □ Change in sexual desire □ Menstrual turbulence □ Acne

27. Who recommended the contraceptive pills?

□Husband □ Family □ Neighbors □ Physician □ Pharmacist □ Nurse □ Midwife □ Media

28. Have you ever stopped using the pills and tried any other contraceptive methods? □ Yes □ No

29. If the answer to the previous answer was yes, what was the reason for trying other methods?

□ Suffered side effects □Pills were not effective □Hard to commit to the pills □ Pills are expensive

□ Pills unavailability □ Other

30. Do you know how the contraceptive pills work? □ Yes □ No

31. If the answer to the previous answer was yes, how do you know?

□ Physician □ Pharmacist □ Media □ Books □Internet □ Other

32. Were the contraceptive pills effective in birth control? □ Yes □ No

33. Did any pregnancies occur during pills usage? □ Yes □ No

34. Do you know what medication can counteract the effect of contraceptive pills? □ Yes □ No

35. Do you think that antibiotics could counteract the effect of contraceptive pills? □ Yes □ No

36. Did the physician/pharmacist give you information on how to preserve the pills effect?

□ Yes □ No

37. Did the physician/pharmacist give you information on how to use the pills? □ Yes □ No
